# Model of Cation Transportation Mediated by High-Affinity Potassium Transporters (HKTs) in Higher Plants

**DOI:** 10.1186/s12575-014-0013-3

**Published:** 2015-01-06

**Authors:** Yi Su, Weigui Luo, Wanhuang Lin, Liying Ma, Mohammed Hunayun Kabir

**Affiliations:** Hunan Provincial Key Laboratory of Phytohormones and Growth Development, Hunan Agricultural University, Changsha, China; Hunan Co-Innovation Center for Utilization of Botanical Functional Ingredients, Changsha, China; Chengnan College, Hunan First Normal University, Changsha, China

**Keywords:** HKT transporters, Cation transport, K^+^/Na^+^ homeostasis, Na^+^-uniport, Na^+^/K^+^-symport

## Abstract

Trk/Ktr/HKT transporters probably were evolved from simple K^+^ channels KcsA. HKT transporters, which mediate Na^+^-uniport or Na^+^/K^+^-symport, maintain K^+^/Na^+^ homeostasis and increase salinity tolerance, can be classified into three subfamilies in higher plants. In this review, we systematically analyzed the characteristics of amino acids sequences and physiological functions of HKT transporters in higher plant. Furthermore, we depicted the hypothetical models of cations selection and transportation mediated by HKT transporters according to the highly conserved structure for the goal of better understanding the cations transportation processes.

## Introduction

Sodium (Na), unlike potassium (K), is not an essential nutrient element for the most of higher plants but may be a beneficial element for some species [1-3]. In higher plants, Na^+^ could act as an osmoticum and temporarily substitute for K^+^ in deficiency or insufficiency of K^+^ [4-6]. Na^+^ is able to stimulate growth of fungi and plants as long as the accumulation and compartmentalization are efficiently controlled below a limited concentration at the cell and tissue levels [6-9]. Excessive Na^+^ in the external environment could lead to the detrimental effects on plant growth, and even cause plant death. The toxic levels did not defined in detail and were supposed to depend on cell types [9], but it is viewed that the cytosolic concentration of Na^+^ should not be higher than 10–30 mM [10]. Additionally, tissue K^+^/Na^+^ ratio is a widely used parameter in discriminating genotypes for salinity tolerance of higher plants [11-20]. Plants can maintain high cytosolic K^+^/Na^+^ ratio through excluding Na^+^ from shoots and accumulating K^+^ in shoots [21-28].

**Figure 1:**
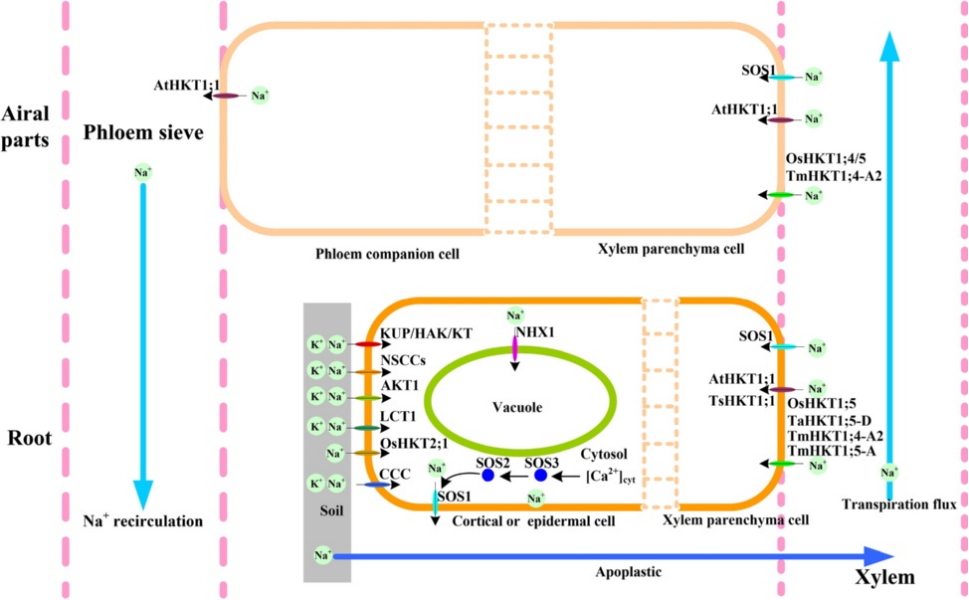
**K** ^**+**^ **/Na** ^**+**^ **homeostasis in higher plants.** Plant root cells generally absorb Na^+^/K^+^ from soil through different channels (NSCCs, AKT1, LCT1, CCC), transporters (KUP/HAK/KT and HKT) and apoplastic. Channel permeations and apoplastic are the main pathways of Na^+^ influx under salt stress. In the SOS pathway Na^+^ crosses the plasma membrane to the apoplast or soil solution and the NHX1 partitions Na^+^ within vacuole and jointly regulate the cytosol Na^+^ concentrations and play a vital role in response to salt stress. AtHKT1;1, OsHKT1;5, TaHKT1;5 and TmHKT1;4/5 retrieve Na^+^ from the xylem into xylem parenchyma cell and prevent the shoot from damage caused by Na^+^ over-accumulation. It is hypothesized that AtHKT1;1 mediates recycling Na^+^ from the shoot to root through removal of Na^+^ from the xylem and loading Na^+^ into the phloem sieves. These processes assure a normal K^+^/Na^+^ homeostasis and maintain a high K^+^/Na^+^ ratio to rescue plants when suffering from salt stress. NSCC, nonselective cation channels; CCC, cation-Cl − co-transporter; LCT, low-affinity cation transporter; SOS1, salt overly sensitive 1; NHX1, Na^+^/H^+^ antiporter 1.

For resisting Na^+^ toxicity, plants developed three mechanisms for salinity tolerance to maintain potassium/sodium homeostasis (Figure 1): 1) Na^+^ exclusion from the shoot, 2) Na^+^ tissue tolerance and 3) osmotic tolerance [29,30]. Till now, a series of transporter systems have been reported which help plants to improve salinity tolerance by inhibiting Na^+^ influx, enhancing Na^+^ efflux, or mediating the sequestration of Na^+^ into the cell vacuoles (Figure 1). Simplified model for mechanisms of K^+^/Na^+^ absorption, recirculation and extrusion by different classes of Na^+^ channels/transporters are shown in Figure 1, such as nonselective cation channels (NSCC) [31-33], cation-Cl^−^ co-transporter (CCC) [34], low-affinity cation transporter (LCT) [35,36], salt overly sensitive 1 ( SOS1) [37-41], Na^+^/H^+^ antiporter NHX1 [42-46] and high affinity potassium transporter (HKT/HAK) [27,28,47-50]. Plant root cells generally take up Na^+^/K^+^ from soil through some channels (NSCCs, AKT1, LCT1 and CCC), transporters (KUP/HAK/KT and HKT) and apoplastic. Channel permeations and apoplastic are the main pathways of Na^+^ influx under salt tress. The SOS pathway mediates efflux of Na^+^ cross the plasma membrane to the soil solution or apoplast. NHX1 partitions Na^+^ within vacuole and jointly regulates the cytosol Na^+^ concentrations. AtHKT1;1, OsHKT1;5, TaHKT1;5 and TmHKT1;4/5 retrieve Na^+^ from the xylem into the xylem parenchyma cell and prevent the shoot from Na^+^ over-accumulation damage. It is hypothesized that AtHKT1;1 mediates recycling of Na^+^ from the shoot to root through removal of Na^+^ from the xylem and loading Na^+^ into the phloem sieves. These processes assure a normal K^+^/Na^+^ homeostasis and also maintain a high K^+^/Na^+^ ratio to rescue plants when suffering salt stress.

HKT transporters belong to a superfamily of Trk/Ktr/HKT and play a vital physiological roles in plants. Plant HKT transporter is a multiple cation uptake system, which can mediate Na^+^ uniport, Na^+^/K^+^-symport and even Mg^2+^/Ca^2+^ permeation. Function of plant HKTs depends on its structure. Therefore, for better understanding how HKT transporters work in higher plants, it is necessary to construct a model of cations uptake mediated by HKT transporters through systematically analyzing the conserved structures of HKTs. In this article we hypothesized a model of cations selection and transportation mediated by HKT transporters.

### Three subfamilies of HKT transporters

**Figure 2:**
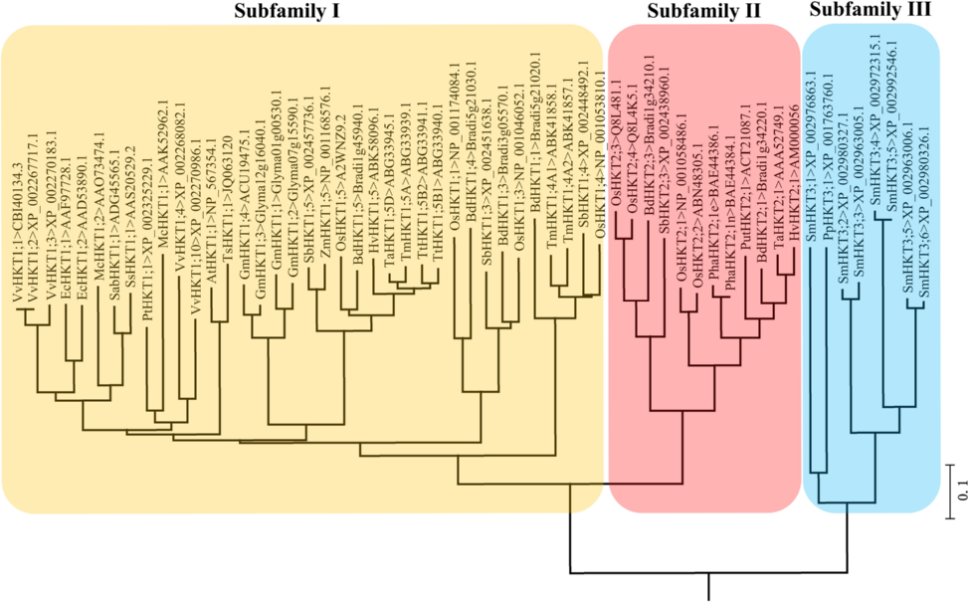
**Phylogenetic analysis of HKT transporters in higher plants.** Subfamily I of HKT transporters are all characterized by “Ser” in the first loop (P_A_). Subfamily II and III have the GlyGlyGlyGly-type characteristic in the amino acid sequences exception of OsHKT2;1. At, *Arabidopsis thaliana*; Ts, *Thellungiella salsuginea*; Pt, *Populus trichocarpa*; Mc, *Mesembryanthemum crystallinum*; Vv, *Vitis vinifera*; Ec, *Eucalyptus camaldulensis*; Sb, *Sorghum bicolor*; Ss, *Suaeda salsa*; Zm, *Zea mays*; Sab, *Salicornia bigelovii*; Os, *Oryza sativa*; Hv, *Hordeum vulgare*; Bd, *Brahypodium distachyom*; Tm, *Triticum monococcum*; Ta, *Triticum aestivum*; Tt, *Triticum timopheevii*; Gm, *Glycine max*; Put, *Puccinellia tenuiflora*; Pha, *Phragmites australis*; Sm, *Selaginella moellendorffii*; Pp, *Physcomitrella patens*.

*HKT* genes encode high affinity potassium transporters in plants and available evidences support that HKTs can be classified into three subfamilies (i.e. subfamily I, subfamily II and subfamily III) according to the phylogenetic analysis based on amino acids of HKTs (Figure 2). Till now, we can retrieve more than one hundred members of HKT transporters from published papers and gene (or protein) databases. The number of HKT transporters in higher plants shows a striking difference among different species. Researchers already identified several *HKT*-like genes in wheat, at least nine in rice, but unique in *Arabidopsis* and *Physcomitrella patens*, since *TaHKT2;1* (originally named *HKT1*) was firstly isolated from wheat roots. It is certain that the monocotyledon contain more HKT transporters than dicotyledon. In addition, HKT transporters of subfamily I were isolated both in the dicotyledon and monocotyledon, but HKTs of subfamily II were isolated only in the monocotyledon. Some HKT transporters have been found in the more primitive higher plants, such as *Selaginella moellendorffii* and *Physcomitrella patens*. Phylogenetic analysis showed that this kind of HKTs should be classed to subfamily III (Figure 2).

Subfamily I members of HKT transporters contain a highly conserved serine (Ser) residue in the first motif MP_A_M, whereas subfamily II members primarily have glycine (Gly) residue with the exception of OsHKT2;1. Mutation from Ser to Gly change the affinity to cations [6,48,51,52]. Subfamily III members are similar to subfamily II with typical GlyGlyGlyGly-type feature. It is hypothesized that subfamily III transporters have the characteristics of K^+^-Na^+^ co-transport but there are few reports [52].

### Structure of HKT transporters in higher plants

HKTs in Plant and Ktr/Trks in bacteria/fungi contain four MPM motifs which might be evolved from simple K^+^ channels KcsA [47,53-62]. Two transmembrane helices (M_1_ and M_2_) and a reentrant loop (P segment) compose the basic motif (MPM motif). Hydropathy plot analysis of Trk/Ktr/HKT systems initially supported a structural model comprising of 8–12 transmembrane segments [53,63-65]. Although every MPM evolved from bacteria KcsA, the four MPM motifs are not simple repeats and they have their own features which determine the selectivity of cations. In fact, the similarity between every two MPMs is less than 30%. Alignment analysis suggested that the fourth MPM motif is the most conserved subunit which is almost similar to KcsA. Bacterial Trk and Ktr are associates with an ion-conducting transmembrane subunit and at least one peripheral regulatory subunit derived from the cleavage of the cytoplasmic C-terminal domain of Trk/Ktr channel [54,55]. Whereas, no regulatory subunit is found in the single amino acid chain systems of fungal Trk and plant HKT transporters till now.

In higher plants, HKT transporters contain some highly conserved amino acid residues which may play a vital function. A Gly or Ser residue in MP_A_M motif (first motif) determines the permeability of K^+^ or Na^+^ [53]. Plant HKTs act as a Na^+^-K^+^ symporter when Gly exists in MP_A_M motif. However, HKT transporters merely show Na^+^ selective-permeability when Gly is substituted by Ser. Therefore, plant HKTs can be classified to SerGlyGlyGly-type and GlyGlyGlyGly-type.

**Figure 3:**
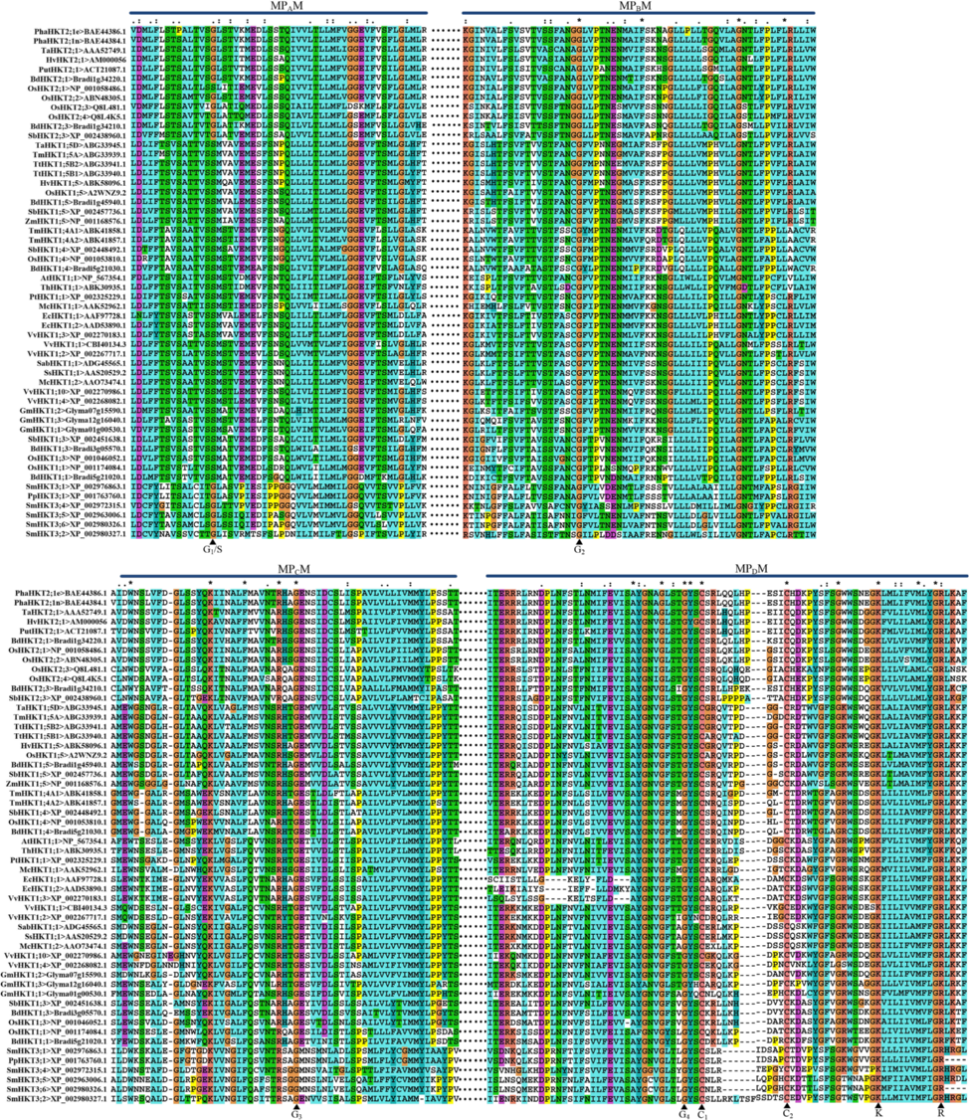
**Multiple alignment of plant HKTs.** The highly conserved signature residues were marked with bold triangle. G: glycine (Gly); S: serine (Ser); C: cysteine (Cys); K: lysine (Lys); R: arginine (Arg).

According to the classical structural model, HKT transporters contain four MPM motifs_,_ which might be evolved from the simple K^+^ KcsA. But, multiple alignments show that the fourth motif MP_D_M is divided into two segments (Figure 3). The fourth signature Gly is located in the first segment and the second segment contains three highly conserved amino acid residues, which are cysteine (Cys), lysine (Lys) and arginine (Arg). Two highly conserved positive amino acid i.e. Arg (R) and Lys (K) residues in the MP_D_M (Figure 3) are not replaceable. These positive residues, which are conserved in many K^+^ channels, contribute to cation transport activity [66]. Kato *et al.* thought that both Lys and Arg residues face towards the ion conducting pore side, and a salt bridge(s) exists between positive residues in MP_D_M motif and conserved negative residues in the pore region to reduce electrostatic repulsion against cation permeation caused by the positive residue(s) [66]. This salt bridge may help stabilize HKTs configuration [66]. Therefore, the MP_D_M motif may be regarded as an independent functional motif because of the separate location and having the quite different role comparing with the other MPM motifs. In addition, it deserves paying close attention to another highly conserved amino acid, cysteine (C) in the fourth motif (C1 and C2 marked with bold triangle in Figure 3).

**Figure 4:**
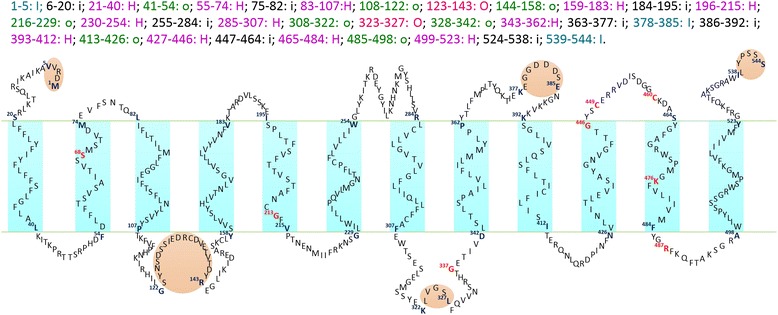
**Structure of AtHKT1;1 transporter.** The letters with red font represent the highly conserved amino acid resides which may play crucial functions on cation selection and transport. H, membrane helix; I, inside loop; i, inside helix tail; O, outside loop; o, outside helix tail.

For obtaining more information about the structure of HKT, the transmembrane structure and hydrophobic features were analyzed through the HMMTOP method (http://www.enzim.hu/hmmtop/index.php). Results of hydrophobicity prediction showed that C-terminal is faced toward intracellular and N-terminal is faced toward extracellular in most of the plant HKT transporters (Table 1). And this result suggests that N-terminal of HKT may be in charge of catching ions but C-terminal is responsible for regulating the permeability. This can explain why HKT transporters mediate a cation from external environment into cytoplasm. The number of transmembrane helixes in HKT transporter ranges from eight to thirteen. There are some loops (the longer part of a sequence outside of the membrane, which can form a domain or a simpler structure) and (or) tails (the elongation of the membrane helix, it can be followed by a loop or another tail, forming a short loop interacting with the outside or inside part of the membrane) between two transmembrane helixes. According to the classical model, the signature Gly (G) and Ser (S) residues were thought to be probably seated in loops between two transmembrane helixes. In fact, the situation may be more complicated because the signature Ser/Gly can be situated in the every structure — membrane helix, inside loop, inside tail, outside loop and outside tail. However, the widespread pattern are: 1) G_1_/S, G_2_ and G_4_ (especially G_1_/S) are mainly located in membrane helix; 2) Nearly all the third glycine residues lie in the helix tail; 3) Two conserved cysteine residues (C_1_ and C_2_) do not lie in the transmembrane helixes except for SmHKT3;1 transporter; 4) For lysine and arginine, if one lies in helix another lies in helix tail with few exceptions (Table 1). In addition, we depicted the typical structure of AtHT1;1 transporter (shown in Figure 4). Except for the twelve transmembrane helixes, AtHT1;1 transporter contains sixteen helix tails but only five loops.

**Table 1:** **The location of N/C terminal and signature residues, and potential transmembrane helix number (THN)**

**Name**	**N-Ter**	**G**_**1**_ **/S**	**G**_**2**_	**G**_**3**_	**G**_**4**_	**C**_**1**_	**C**_**2**_	**K**	**R**	**THN**	**C-Ter**
AtHKT1;1	inside	H	H	o	H	i	i	H	i	12	inside
ThHKT1;1	inside	H	H	o	H	O	o	H	i	10	inside
OsHKT1;1	outside	I	H	o	i	i	i	i	H	10	outside
EcHKT1;1	inside	H	H	o	H	O	o	H	i	12	inside
PtHKT1;1	outside	H	H	i	H	o	o	H	i	11	inside
McHKt1;1	inside	H	H	i	H	o	o	H	i	12	inside
McHKT1;2	inside	I	H	o	H	O	o	H	i	10	inside
OsHKT1;3	outside	i	H	o	H	i	i	H	o	10	outside
TmHKT1;4A1	inside	o	H	i	H	o	o	H	i	10	inside
TmHKT1;4A2	inside	o	H	i	H	o	o	H	i	10	inside
SbHKT1;4	outside	o	H	i	H	o	o	H	i	11	inside
OsHKT1;4	outside	H	O	i	H	o	o	H	i	11	inside
TmHKT1;5	outside	H	H	i	H	o	o	H	i	13	inside
TaHKT1;5	outside	H	O	o	H	O	o	H	i	9	inside
TtHKT1;5B2	outside	H	O	o	H	O	o	H	i	9	inside
HvHKT1;5	inside	o	H	i	o	o	o	o	H	10	inside
ZmHKT1;5	inside	H	H	i	o	o	o	o	H	10	inside
OsHKT1;5	outside	H	O	i	O	o	O	O	O	10	outside
OsHKT2;1	outside	H	H	o	o	i	i	H	o	12	outside
PhaHKT2;1e	outside	H	H	o	o	O	O	o	H	11	inside
TaHKT2;1	inside	H	O	i	o	o	o	o	H	12	inside
HvHKT2;1	outside	H	H	i	o	o	o	o	H	11	inside
PutHKT2;1	inside	H	H	i	o	o	o	o	H	12	inside
PhaHKT2;1n	outside	H	H	o	o	O	O	o	H	11	inside
OsHKT2;2	outside	H	O	o	O	O	O	O	O	8	outside
SbHKT2;3	outside	H	O	o	H	O	o	H	i	9	inside
OsHKT2;3	outside	H	O	i	H	o	o	H	i	11	inside
OsHKT2;4	inside	H	H	i	H	o	o	H	i	12	inside
PpHKT3;1	outside	H	H	H	O	O	O	O	O	8	outside
SmHKT3;1	inside	o	O	o	H	H	i	H	o	9	outside
SmHKT3;2	inside	o	H	i	H	o	o	H	i	10	inside
SmHKT3;4	inside	H	H	o	H	o	o	H	i	11	outside
SmHKT3;5	outside	H	H	i	H	i	i	H	o	11	inside

*Abbreviations* represent: H, membrane helix; I, inside loop; i, inside helix tail; O, outside loop; o, outside helix tail. G: glycine (Gly); S: serine (Ser); C: cysteine (Cys); K: lysine (Lys); R: arginine (Arg).

There are two interesting exceptions OsHKT2;2 and PpHKT3;1 which functions did not follow the universal principles. OsHKT2;2 share 91% identity with OsHKT2;1 in amino acid sequences. OsHKT2;1 mediates Na^+^ uptake both in plant and heterologous systems. In contrast, OsHKT2;2, previously found to be a pseudogene in Nipponbare rice (*japonica* rice) but not in *indica* rice [60]. Furthermore, at millimolar Na^+^ concentrations, OsHKT2;2 mediated Na^+^ influx into plant cells without adding extra cellular K^+^ [67]. PpHKT3;1 (originally named PpHKT1) transporter is the unique one in *Physcomitrella patens* and characterizes with Gly in the first motif. However, PpHKT1 transporter mediated K^+^ and Na^+^ influx but not high-affinity Na^+^ uptake because *Pphkt1* mutant plants maintain normal K^+^ and Na^+^ influx [52]. Screening of the transmembrane and topology structure, we found that OsHKT2;2 and PpHKT1 transporter only contain eight transmembrane helixes, and signature conserved residues are mainly located outside of the cytomembrane (Table 1). These different structure characteristics may be the reason for the different phenomena on cations uptake.

### Various cations transport characteristics based on amino acid sequences in higher plants

**Figure 5:**
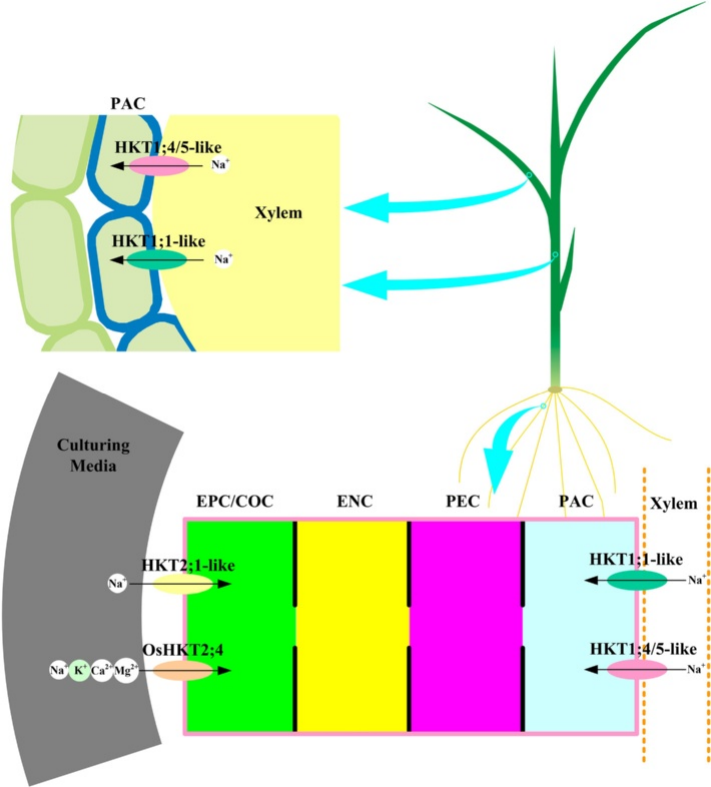
**Functions of HKT transporters in higher plants.** The transporters of HKT2;1-like, including OsHKT2;1, HvHKT2;1 and TaHKT2;1, mediate Na^+^ uptake from culturing media merely in K^+^-starved environments. HKT transporters, such as AtHKT1;1, OsHKT1;5, TaHKT1;5-D, TmHKT1;4-A2 and TmHKT1;5-A are involved in Na^+^ exclusion from xylem to xylem parenchyma cell in order to minimize the accumulation of Na^+^ in the shoot through the transpiration stream, and this is the key process for salinity tolerance of plants. OsHKT2;4 is a very special member in HKT family, and it is not only conducts as a transporter of Na^+^-K^+^ symport but also mediate Ca^2+^ (maybe other divalent cations) uptake like a cation channel. Cell types depicted include: epidermal cell (EPC), cortical cell (COC), endodermis cell (ENC), pericycle cell (PEC), parenchyma cell (PAC).

SerGlyGlyGly-type characteristic determines the HKT as a Na^+^-uniporter. All HKT members in subfamily I are characterized by SerGlyGlyGly. Either in dicotyledons or monocotyledons, most of the HKT members of subfamily I have been looked upon as Na^+^-specific transporters [60]. In *Arabidopsis* genome, *AtHKT1;1* is the unique member, which is mainly expressed in xylem parenchyma cells [27,48,68,69]. AtHKT1;1 mediates Na^+^ but small degree K^+^ influx into cells when heterologously expressed in *Xenopus laevis oocytes* and *Saccharomyces cerevisiae* [47]. In addition all the identified mutants of *AtHKT1;1* have been found to be salt sensitive and Na^+^ over-accumulation in aerial organs but Na^+^ under-accumulation in roots [27,48,68,70,71]. Thus AtHKT1 decreases Na^+^ concentration in the transpiration stream and increase salinity tolerance following two patterns: 1) Na^+^ retrieval in the root through unloading sodium directly from the xylem sap to xylem parenchyma cells, and 2) Na^+^ recirculation in shoot through removal of Na^+^ from the xylem sap and then transporting Na^+^ from phloem companion cells into the phloem sieves. Both pathways can effectively minimize the over-accumulation of Na^+^ in shoot and thus protect the leaves from salt damage when suffering from salt stress (Figure 5). Additionally, earlier investigations indicated that over-expression of *AtHKT1;1* in specific cell types could modify Na^+^ transport process with the reduction of shoot Na^+^ accumulation and thus improve salinity tolerance. Møller *et al*. [72] revealed that Na^+^ accumulation was decreased from 37 to 64% in shoot because of increased influx of Na^+^ into root stellar cells when overexpressed *AtHKT1;1* in the mature root stele [72]. Rice obtained higher Na^+^ exclusion and salinity tolerance when *AtHKT1;1* was expressed in the root cortical and epidermal cells [73]. These results have implied that the alteration of a specific Na^+^ transport process in specific cell types leads to a decrease of shoot Na^+^ accumulation, which is a mechanism of salt stress in higher plants [73,74]. In the various mechanisms of salt tolerance (mentioned above), osmotic tolerance or tissue tolerance, mediated by other channels and transporters, might be more important in enabling *Arabidopsis* plants to grow in saline conditions than Na^+^ exclusion [75-77].

EcHKT1;1/2 from *Eucalyptus camaldulensis* can mediate both Na^+^ and K^+^ influx when expressed in *Xenopus oocytes* [56,78]. McHKT1;1/2, characterized from *Mesembryanthemum crystallinum*, can conduct K^+^-Na^+^ co-transport or K^+^ uptake in heterologous expression systems [59]. McHKT1;1 and TsHKT1;2 were up-regulated after a sudden increase of external NaCl [59,79,80]. TaHKT1;5-D, TmHKT1;4-A2(Nax1) and TmHKT1;5-A(Nax2), AtHKT1;1 homologs, mediate Na^+^ uptake in xylem parenchyma cells and Na^+^ loading into the phloem sap, thereby improve the salt tolerance [81-87]. OsHKT1;5 (OsSKC1), located in parenchyma cells surrounding the xylem vessels, is likely to function in loading Na^+^ from the xylem into the xylem parenchyma cells [26]. TmHKT1;4-A2 expressed in roots and leaf sheaths of a salt-tolerant durum wheat line 149, and mediated Na^+^ influx from xylem sap to the xylem parenchyma cells [82,88]. TaHKT1;5-D and TmHKT1;5-A mediated Na^+^ transportation from roots xylem then and maintained a high K^+^-to-Na^+^ ratio in the leaves [81,84,86]. In addition, functional analysis in *Xenopus laevis oocytes* revealed that OsHKT1;1 and OsHKT1;3 are permeable to Na^+^ only, but are strongly different in terms of affinity and direction of transport (inward only or reversible) [60,89].

OsHKT2;1 is the unique member characterized by SerGlyGlyGly in subfamily II. OsHKT2;1 is mainly expressed in cortical and endodermal cells of roots and vascular bundle regions of leaves [6]. OsHKT2;1 displays three models of ion selectivity according to external K^+^ and/or Na^+^ in heterologous expression systems i.e. OsHKT2;1 acts as 1) Na^+^-K^+^ co-transporter at submillimolar level of external Na^+^ and K^+^, 2) Na^+^ uniport when the external Na^+^ content is within or above the millimolar range or when the external K^+^ is in the submillimolar range 3) and nonconductive states within the millimolar to 10 mM range of external K^+^ [89]. The *in vivo* functional analysis demonstrated that Na^+^ enhanced growth of rice under K^+^ starvation conditions, and OsHKT2;1 is the central transporter for nutritional Na^+^ uptake in case of K^+^-starved rice roots [6,67].

GlyGlyGlyGly-type feature decides the Na^+^/K^+^-symport. The first motif MP_A_M contains a Gly residue in all the HKT members of subfamily II with the exception of OsHKT2;1 (previously named OsHKT1) [45,65,74,90,91]. In wheat and barley roots, TaHKT2;1(TaHKT1) and HvHKT2;1 (HvHKT1) mediate Na^+^ uptake at K^+^-starved situation [61,92]. OsHKT2;2 is one of the typical HKT transporters of subfamily II with GGGG-type amino acids sequence in rice, which has been found to be permeable to both K^+^ and Na^+^ [57,67,91]. Kader *et al*. [93] reported that expression of the *OsHKT2;2* gene is detected in the phloem of leaves when treated with 150 mM NaCl [93]. TaHKT2;1 in wheat, PhaHKT2;1 in *Phragmites australis* and HvHKT2;1/2 in Tibetan wild barely have been shown at least two transport modes in heterologous expression systems, K^+^-Na^+^ co-uptake and Na^+^ influx at high Na^+^ concentrations [66,69,94-97]. However, OsHKT2;4 showed different cation selectivity. OsHKT2;4 transporter, unlike with the other subfamily II HKT transporters, mediates robust inward K^+^ currents even without the addition of extracellular Na^+^ in heterologous expression systems, and also functions as a Mg^2+^ and Ca^2+^ permeable channel in the absence of competing K^+^ ions [98-100]. This implies that OsHKT2;4 is likely to be more important in K^+^ homeostasis as a K^+^ transporter/channel than a Na^+^-K^+^ co-transporter [99,100].

HKT transporters in subfamily III are similar to subfamily II members with the characteristics of GlyGlyGlyGly, but their functions are uncertain. The phylogenetic analysis reveals that all the HKTs of subfamily I and II are belong to flowering plants, but the remainders are collected from some primitive higher plants such as PpHKT in *Physcomitrella patens* and SmHKTs in *Selaginella moellendorffii* (Figure 2). Thereby, these HKTs may be categorized into subfamily III because they are more identical with the ancestral transporters Trk in yeast. *PpHKT3;1* (originally named PpHKT1) which was identified as a unique *HKT* gene in *Physcomitrella patens* [5,101,102]. Regretfully, *Pphkt1* mutant plants maintained normal K^+^ and Na^+^ influx and thus PpHKT1 transporter did not mediate high-affinity Na^+^ uptake [52]. Consequently, the functions of subfamily III HKTs still remain unknown, and further studies are imperative.

### Cation selection model mediated by plant HKT transorters

In bacteria, archaea, fungi and plants the Trk/Ktr/HKT transporters are the key factors of osmotic regulation, pH homeostasis and resistance to drought and high salinity [16,72-74,103]. These cation transporters are functionally diverse i.e. Na^+^ uniporter, Na^+^/K^+^ symporter and even divalent cation transporter [9,15-20,50,104,105]. However, some key informations are still unclear: 1) How do HKTs specifically catch the cations? 2) How do the energy transfers and exchanges since the K^+^/Na^+^ transport mediated by HKTs is an active pathway? 3) What is the mechanism(s) to monitor K^+^/Na^+^ concentration to regulate gene expression and transport activities? The crystal structure of a Ktr K^+^ transporter from *Bacillus subtilis* and TrkH from *Vibrio parahaemolyticus* showed that Ktr and TrkH were resembled K^+^ channel [106,107]. KtrB and TrkH assemble with KtrA and TrkA respectively. The activities of Trk and Ktr are upregulated by ATP respectively via TrkA and KtrA [106,107]. This suggests a mechanism for how ATP activates the activity of TrkH and Ktr by inducing conformational changes.

**Figure 6:**
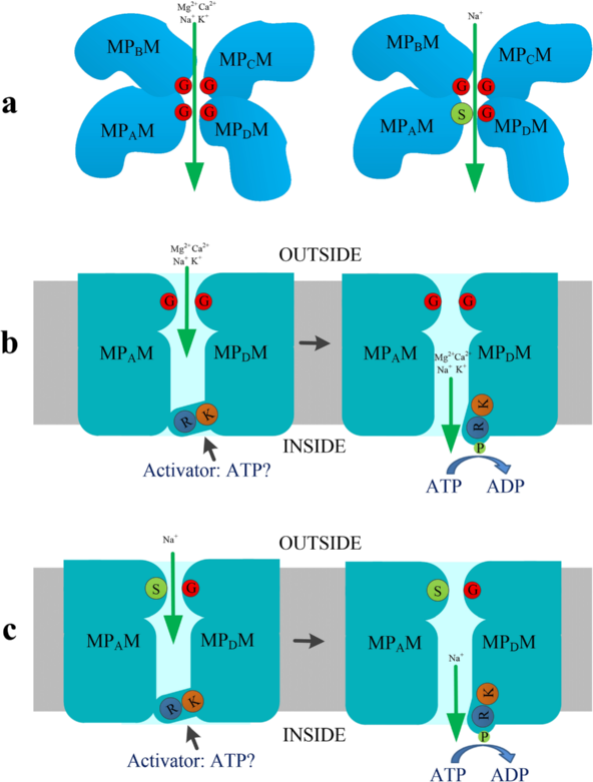
**Model of cation trapping and selection of plant HKTs. a)** Four glycine (G) residues form a trap space and allow Na^+^, K^+^, Mg^2+^ and Ca^2+^ across. Serine (S) and three glycine (G) residues form a trap space and allow Na^+^ across. **b)** and **c)** Positive arginine (R) and lysine (K) residues form a cation barrier to stop cation across.

Additionally, two highly conserved positively charged arginine (R) and lysine (K) residues are present in the MP_D_M helix of plant HKT transporters (Figure 3). Lacking of arginine (R) or lysine (K) could cause the functional loss of HKT transporters (Figure 6). Cation transporters require a barrier to prevent free diffusion of ions along their electrochemical gradient, and it is possible that the positive residues within the transporter’ pore could help to regulate its activities. Individual replacement of positively charged residues in the MP_D_M helices with glutamine (Gln) did not abolish the cation uptake activity of plant HKTs, indicating that exchange of one of the positively charged residues in the MP_D_M helix of plant HKTs with a hydrophilic residue can be tolerated [66]. Replacing of two or more positively charged residues with glutamine caused a considerable loss of activity in TaHKT2;1 [66]. It is hypothesized that lysine and arginine residues form a salt bridge(s) in the MP_D_M to help to stabilize HKTs configuration [66]. Here we are suggesting another model for explain how the positive arginine and lysine work (Figure 6b and c). Generally, the arginine and lysine are positively charged and the electrostatic repulsion will refuse cation permeation from pore folded by HKT into the cell. The MP_D_M helix with positive residues can be looked as a cation barrier or switch. A certain activator would arouse the conformational change of barrier and then the switch will turn on (Figure 6b and c). ATP is a general energy driving ion transportation on membrane and this process is companied with the transporter phosphorylation which will trigger structural change and ion permeation [106-108]. Plant HKTs may be activated by this manner in a view of the universal mechanism about molecular switch mediated by phosphorylation. Probably, the highly conserved hydroxyl amino acid resides, such as serine (S), threonine (T) and tyrosine (Y) in the MP_D_M (S, T and Y rich region in Figure 3), contribute to this phosphorylation process. However, no evidence indicates that HKTs are related with activity of ATPase up to date. Researchers still need to keep searching for which ATPase mediates the phosphorylation process.

Considering all the former evidences, we think that plant HKT transporter may be able to perform ion transportation following this hypothesis cation selection model (Figure 6). Plant HKTs include SerGlyGlyGly-type and GlyGlyGlyGly-type transport. SerGlyGlyGly-type HKTs mainly mediate Na^+^ uniport but GlyGlyGlyGly-type HKTs are diversified characteristics for cations selectivity. This class of HKTs can mediate Na^+^-K^+^symport and even divalent cations transport [57,67,100]. According to the helical wheel model structure [54], the four signature residues form a space which works as a cation trapping site (Figure 6). Gly is the smallest amino acid and Ser has polarity. Therefore, the space assembled by GlyGlyGlyGly is more flexible than SerGlyGlyGly. The more flexible space lets plant HKTs to catch more type of cations, such as divalent Mg^2+^/Ca^2+^ and bigger K^+^. As an activator, ATP can drive structure conversion of plant HKTs and the switch on (Figure 6). That process possibly accompanies with phosphorylation of serine, threonine or (and) tyrosine in the MP_D_M motif. This cation selection model could be used to explain why GGGG-type HKTs show more complicated features on cation selectivity than SGGG-type.

### Hypothesis on HKT polymer

**Figure 7:**
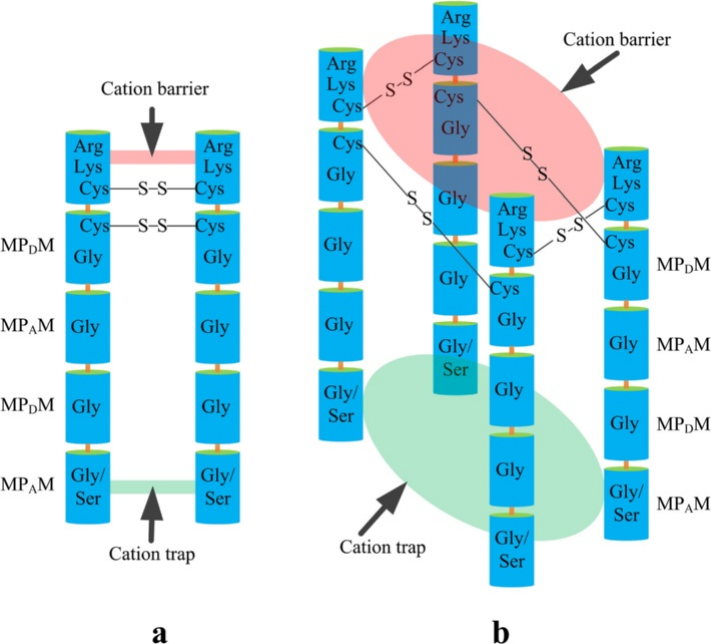
**Hypothesis of HKT polymer model.** Two highly conserved cysteine (Cys) residues form disulfide bonds to help HKTs to assemble a dimer or tetramer and stabilize the structures. Positive arginine (Arg) and lysine (Lys) residues form a cation barrier to stop cation across. Two/four serine (Ser) or glycine (Gly) residues form a cation trap for plant HKTs specifically selecting cation. **a)** dimer model and **b)** tetramer model.

TrkH transporter may play a role of K^+^ transport through assembling to tetramer [54,106]. Interestingly, there are two highly conserved cysteine residues (C1 and C2 marked with bold triangle in Figure 3) in MP_D_M motif according to the multiple alignments. These conserved cysteine residues (C_1_ and C_2_) mainly lie in helix tail but not transmembrane helixes (Table 1 and Figure 4). Functional complementation experiments in yeast trk1trk2 mutant and Na^+^ hypersensitive mutant suggests that these two cysteine residues are indispensable (Figure 6). In tissues of organisms, a crucial function of cysteine residues is to cross link of proteins or protein subunits through disulfide bonds. This indicates that chains of HKT may be able to assemble a dimer or a tetramer through the two cysteine residues (Figure 7). In this model, cysteine residues can stabilize the structure configuration of HKTs. Positive resides of two or four group of arginine (Arg) and lysine (Lys) can make a cation barrier or switch which usually turn off, but the switch will be turned on when an activator binds to the MP_D_M motif (Figure 6). Additionally, more Gly or Ser residues will be involved in the forming of cation trap/space according to the HKT polymer model, and this situation provides plant HKTs with more flexibility on cations selection.

### Conclusions and future directions

Trk/Ktr/HKT were generally thought to be evolved from the bacterial KcsA K^+^ channel [109] and contain four conserved MPM motifs [55,58,65,110]. Results of phylogenetic analysis showed that plant HKTs can be classified to three subfamilies. Subfamily I was characterized by SerGlyGlyGly but subfamily II and III were GlyGlyGlyGly-type HKTs exception of OsHKT2;1. Till now, the physiological functions of HKTs in higher plant have been well understood. GGGG-type HKTs are Na^+^-K^+^ co-transporters, and SGGG-type HKTs present Na^+^ specific-selectivity.

Former model about cation selectivity of plant HKTs emphasizes on the vital function of first motif MP_A_M based on the diversity of signature residues. In fact, there are some key questions still unsolved. Firstly, it is needed to be further clarified in the details of molecular mechanism that how plant HKTs specifically trap a certain cation. Secondly, what energy materials take part in the active transport mediated by plant HKTs, and how the energy transfers and exchanges. We supposed that the fourth motif MP_D_M also have same importance for cation permeation conducted by plant HKTs since this motif is more conserved than other MPMs. Highly conserved positive residues arginine and lysine in MP_D_M may be a cation barrier/switch which prevents cation permeation along the pore folded by HKT into intracellular. A certain activator, most probably ATP, binds to MP_D_M motif (or another motif) and drives the conformational change of HKTs, and then the cation switch turned on (Figure 7). Moreover, protein chains can be cross-linked through disulfide bonds condensed by cysteine resides. Interestingly, there are exactly two highly conserved cysteine residues in motif MP_D_M. Therefore, we hypothesize that plant HKTs possibly assemble to a dimer or tetramer through the two conserved Cys residues based on tetrameric model for the Trk family of symporters [54,106]. However, the model of cations transport through HKT transporters still need be supported by more experimental evidences i.e. 1) functional identification about specific amino acid mutations, 2) high-resolution distribution of HKT in membrane, 3) determination of chemicals related to energy transformation 4) and especially crystal structure interpretation of plant’ HKTs.

## References

[1] Sobbarao GV, Ito O, Berry WL, Wheeler RM (2003). Sodium-A functional plant nutrient. Crit Rev Plant Sci.

[2] Kronzucker HJ, Coskun D, Schulze LM, Wong JR, Britto DT (2013). Sodium as nutrient and toxicant. Plant Soil.

[3] Maathuis FJ (2014). Sodium in plants: perception, signalling, and regulation of sodium fluxes. J Exp Bot.

[4] Maathuis FJM, Sanders D (1993). Energization of potassium uptake in *Arabidopsis thaliana*. Planta.

[5] Rodríguez-Navarro A (2000). Potassium transport in fungi and plants. BBA-Proteins Proteom.

[6] Horie T, Costa A, Kim TH, Han MJ, Horie R, Leung HY (2007). Rice OsHKT2,1 transporter mediates large Na^+^ influx component into K^+^-starved roots for growth. EMBO J.

[7] Camacho MR, Rodríguez-Navarro A (1981). Potassium requirements of *Saccharomyces cerevisiae*. Curr Microbiol.

[8] Matoh T, Murata S (1990). Sodium stimulates growth of Panicum coloratum through enhanced photosynthesis. Plant Physiol.

[9] Munns R, Tester M (2008). Mechanisms of salinity tolerance. Annu Rev Plant Biol.

[10] Ben Amar S, Brini F, Sentenac H, Masmoudi K, Véry AA (2014). Functional characterization in *Xenopus oocyte*s of Na^+^ transport systems from durum wheat reveals diversity among two HKT1;4 transporters. J Exp Bot.

[11] Gorham J, Bridges J, Dubcovsky J, Dvorak J, Hollington PA, Luo MC (1997). Genetic analysis and physiology of a trait for enhanced K^+^/Na^+^ discrimination in wheat. New Phytol.

[12] Santa-Maria GE, Epstein E (2001). Potassium/sodium selectivity in wheat and the amphiploid cross X *Lophopyrum elongatum*. Plant Sci.

[13] Munns R, James RA (2003). Screening methods for salinity tolerance: a case study with tetraploid wheat. Plant Soil.

[14] Colmer TD, Flowers TJ, Munns R (2006). Use of wild relatives to improve salt tolerance in wheat. J Exp Bot.

[15] Rodrigues RFC, Silva NE, Ferreira-Silva L, Voigt LE, Viégas AR, Silveira AGJ (2013). High K^+^ supply avoids Na^+^ toxicity and improves photosynthesis by allowing favorable K^+^: Na^+^ ratios through the inhibition of Na^+^ uptake and transport to the shoots of *Jatropha curcas* plants. J Plant Nutr Siol Sci.

[16] Bafeel SO (2013). Phylogeny of the plant salinity tolerance related HKT genes. In J Biol.

[17] Asins MJ, Villalta I, Aly MM, Olias R, Alvarez De Morales P, Huertas R (2013). Two closely linked tomato HKT coding genes are positional candidates for the major tomato QTL involved in Na^+^/K^+^ homeostasis. Plant Cell Environ.

[18] Hasegawa PM (2013). Sodium (Na^+^) homeostasis and salt tolerance of plants. Environ Exp Bot.

[19] Wakeel A (2013). Potassium-sodium interactions in soil and plant under saline-sodic conditions. J Plant Nutr Siol Sci.

[20] Wang M, Zheng Q, Shen Q, Guo S (2013). The critical role of potassium in plant stress response. Int J Mol Sci.

[21] Flowers TJ, Lauchli A (1983). Sodium versus potassium: substitution and compartmentation. Encyclopedia Plant Physiol.

[22] Schachtman DP, Bloom AJ, Dvořák J (1989). Salt-tolerant *Triticum* × *Lophopyrum* derivatives limit the accumulation of sodium and chloride ions under saline stress. Plant Cell Environ.

[23] Dubcovsky J, María GS, Epstein E, Luo MC, Dvorák J (1996). Mapping of the K^+^/Na^+^ discrimination locus *Kna1* in wheat. Biomed Life Sci.

[24] Apse MP, Aharon GS, Snedden WA, Blumwald E (1999). Salt tolerance conferred by overexpression of a vacuolar Na^+^/ H^+^ antiport in *Arabidopsis*. Science.

[25] Shi H, Quintero FJ, Pardo JM, Zhu JK (2002). The putative plasma membrane Na^+^/H^+^ antiporter SOS1 controls long distance Na^+^ transport in plants. Plant Cell.

[26] Ren ZH, Gao JP, Li LG, Cai XL, Huang W, Chao DY (2005). A rice quantitative trait locus for salt tolerance encodes a sodium transporter. Nat Genet.

[27] Horie T, Motoda J, Kubo M, Yang H, Yoda K, Horie R (2005). Enhanced salt tolerance mediated by AtHKT1 transporter-induced Na^+^ unloading from xylem vessels to xylem parenchyma cells. Plant J.

[28] Coskun D, Britto DT, Li M, Oh S, Kronzucker HJ (2013). Capacity and plasticity of potassium channels and high-affinity transporters in roots of barley and *Arabidopsis*. Plant Physiol.

[29] Rajendran K, Tester M, Roy SJ (2009). Quantifying the three main components of salinity tolerance in cereals. Plant Cell Environ.

[30] Flowers TJ, Galal HK, Bromham L (2010). Evolution of halophytes: multiple origins of salt tolerance in land plants. Funct Plant Biol.

[31] Demidchik V, Tester M (2002). Sodium fluxes through nonselective cation channels in the plasma membrane of protoplasts from *Arabidopsis* roots. Plant Physiol.

[32] Gobert A, Park G, Amtmann A, Sanders D, Maathuis FJM (2006). Arabidopsis thaliana cyclic nucleotide gated channel 3 forms a non-selective ion transporter involved in germination and cation transport. J Exp Bot.

[33] Guo KM, Babourina O, Christopher DA, Borsic T, Rengel Z (2010). The cyclic nucleotide-gated channel AtCNGC10 transports Ca^2+^ and Mg^2+^ in *Arabidopsis*. Physiol Plantarum.

[34] Hebert SC, Mount DB, Gamba G (2004). Molecular physiology of cation-coupled Cl^−^ cotransport: the SLC12 family. Pflugers Arch.

[35] Schachtman DP, Kumar R, Schroeder JI, Marsh EL (1997). Molecular and functional characterization of a novel low affinity cation transporter (LCT1) in higher plants. Proc Natl Acad Sci U S A.

[36] Amtmann A, Fischer M, Marsh EL, Stefanovic A, Sanders D, Schachtman DP (2001). The wheat cDNA *LCT1* generates hypersensitivity to sodium in a salt-sensitive yeast strain. Plant Physiol.

[37] Shi H, Ishitani M, Kim C, Zhu JK (2000). The *Arabidopsis thaliana* salt tolerance gene *SOS1* encodes a putative Na^+^/H^+^ antiporter. Proc Natl Acad Sci U S A.

[38] Shi H, Lee BH, Wu SJ, Zhu JK (2003). Overexpression of a plasma membrane Na^+^/H^+^ antiporter gene improves salt tolerance in *Arabidopsis thaliana*. Nat Biotechnol.

[39] Blumwald E, Aharon GS, Apse MP (2000). Sodium transport in plant cells. BBA-Proteins Proteom.

[40] Pardo JM (2010). Biotechnology of water and salinity stress tolerance. Curr Opin Biotechnol.

[41] Pardo JM, Cubero B, Leidi EO, Quintero FJ (2006). Alkali cation exchangers: roles in cellular homeostasis and stress tolerance. J Exp Bot.

[42] Apse MP, Sottosanto JB, Blumwald E (2003). Vacuolar cation/H^+^ exchange, ion homeostasis, and leaf development are altered in a T-DNA insertion mutant of *AtNHX1*, the *Arabidopsis* vacuolar Na^+^/H^+^ antiporter. Plant J.

[43] Sottosanto JB, Gelli A, Blumwald E (2004). DNA array analyses of *Arabidopsis thaliana* lacking a vacuolar Na^+^/H^+^ antiporter: impact of AtNHX1 on gene expression. Plant J.

[44] Sottosanto JB, Saranga Y, Blumwald E (2007). Impact of AtNHX1, a vacuolar Na^+^/H^+^ antiporter, upon gene expression during short- and long-term salt stress in *Arabidopsis thaliana*. BMC Plant Biol.

[45] Kronzucker HJ, Britto DT (2011). Sodium transport in plants: a critical review. New Phytol.

[46] Gálvez FJ, Baghour M, Hao G, Cagnac O, Rodríguez-Rosales MP, Venema K (2012). Expression of LeNHX isoforms in response to salt stress in salt sensitive and salt tolerant tomato species. Plant Physiol Bioc.

[47] Uozumi N, Kim EJ, Rubio F, Yamaguchi T, Muto S, Tsuboi A (2000). The *Arabidopsis HKT1* gene homolog mediates inward Na^+^ currents in *Xenopus laevis oocytes* and Na^+^ uptake in *Saccharomyces cerevisiae*. Plant Physiol.

[48] Benito B, Haro R, Amtmann A, Cuin TA, Dreyer I (2014). The twins K and Na in plants. J Plant Physiol.

[49] Gierth M, Mäser P, Schroeder JI (2005). The potassium transporter AtHAK5 functions in K^+^ deprivation-induced high-affinity K^+^ uptake and AKT1 K^+^ channel contribution to K^+^ uptake kinetics in *Arabidopsis* roots. Plant Physiol.

[50] Benito B, Garciadeblás B, Rodriguez-Navarro A (2012). HAK transporters from *Physcomitrella patens* and *Yarrowia lipolytica* mediate sodium uptake. Plant Cell Physiol.

[51] Horie T, Hauser F, Schroeder JI (2009). HKT transporter-mediated salinity resistance mechanisms in *Arabidopsis* and monocot crop plants. Trend Plant Sci.

[52] Haro R, Banuelos MA, Rodríguez-Navarro A (2010). High-affinity sodium uptake in land plants. Plant Cell Physiol.

[53] Schachtman DP, Schroeder JI (1994). Structure and transport mechanism of a high-affinity potassium uptake transporter from higher plants. Nature.

[54] Durell SR, Guy HR (1999). Structural models of the KtrB, TrkH, and Trk1,2 symporters based on the structure of the KcsA K^+^ channel. Biophys J.

[55] Durell SR, Hao Y, Nakamura T, Bakker EP, Guy R (1999). Evolutionary relationship between K^+^ channels and symporters. Biophys J.

[56] Fairbairn DJ, Liu WH, Schachtman DP, Gomez-Gallego S, Day SR, Teasdale RD (2000). Characterisation of two distinct HKT1-like potassium transporters from *Eucalyptus camaldulensis*. Plant Mol Biol.

[57] Horie T, Yoshida K, Nakayama H, Yamada K, Oiki S, Shinmyo A (2001). Two types of HKT transporters with different properties of Na^+^ and K^+^ transport in *Oryza sativa*. Plant J.

[58] Kato Y, Sakaguchi M, Mori Y, Saito K, Nakamura T, Bakker EP (2001). Evidence in support of a four transmembrane-pore-transmembrane topology model for the *Arabidopsis thaliana* Na^+^/K^+^ translocating AtHKT1 protein, a member of the superfamily of K^+^ transporters. Proc Natl Acad Sci U S A.

[59] Su H, Balderas E, Vera-Estrella R, Golldack D, Quigley F, Zhao CS (2003). Expression of the cation transporter McHKT1 in a halophyte. Plant Mol Biol.

[60] Garciadeblás B, Senn ME, Banuelos MA, Rodríguez-Navarro A (2003). Sodium transport and HKT transporters: The rice model. Plant J.

[61] Haro R, Bañuelos MA, Senn ME, Barrero-Gil J, Rodríguez-Navarro A (2005). HKT1 mediates sodium uniport in roots. Pitfalls in the expression of HKT1 in yeast. Plant Physiol.

[62] Shao Q, Zhao C, Han N, Wang BS (2008). Cloning and expression pattern of SsHKT1 encoding a putative cation transporter from halophyte *Suaeda salsa*. Mitochondr DNA.

[63] Gaber RF, Styles CA, Fink GR (1988). *TRK1* encodes a plasma membrane protein required for high-affinity potassium transport in *Saccharomyces cerevisiae*. Mol Cell Biol.

[64] Liu WH, Schachtman DP, Zhang W (2000). Partial deletion of a loop region in the high affinity K^+^ transporter HKT1 changes ionic permeability leading to increased salt tolerance. J Biol Chem.

[65] Corratgé-Faillie C, Jabnoune M, Zimmermann S, Véry AA, Fizames C, Sentenac H (2010). Potassium and sodium transport in non-animal cells: the Trk/Ktr/HKT transporter family. Cell Mol Life Sci.

[66] Kato N, Akai M, Zulkifli L, Matsuda N, Kato Y, Goshima S (2007). Role of positively charged amino acids in the M2D transmembrane helix of Ktr/Trk/HKT type cation transporters. Channel.

[67] Berthomieu P, Conejero G, Nublat A, Brackenbury WJ, Lambert C, Savio C (2003). Functional analysis of AtHKT1 in *Arabidopsis* shows that Na^+^ recirculation by the phloem is crucial for salt tolerance. EMBO J.

[68] Rus A, Lee BH, Munoz-Mayor A, Sharkhuu A, Miura K, Zhu JK (2004). AtHKT1 facilitates Na^+^ homeostasis and K^+^ nutrition in planta. Plant Physiol.

[69] Davenport RJ, Muñoz-Mayor A, Jha D, Essah PA, Rus A, Tester M (2007). The Na^+^ transporter AtHKT1 controls xylem retrieval of Na^+^ in *Arabidopsis*. Plant Cell Environ.

[70] Møller IS, Gilliham M, Jha D, Mayo GM, Roy SJ, Coates JC (2009). Shoot Na^+^ Exclusion and increased salinity tolerance engineered by cell type-specific alteration of Na^+^ transport in *Arabidopsis*. Plant Cell.

[71] Jha D, Shirley N, Tester M, Roy SJ (2010). Variation in salinity tolerance and shoot sodium accumulation in *Arabidopsis* ecotypes linked to differences in the natural expression levels of transporters involved in sodium transport. Plant Cell Environ.

[72] Plett D, Safwat G, Gilliham M, Møller IS, Roy S, Stuart R (2010). Improved salinity tolerance of rice through cell type-specific expression of AtHKT1,1. PLoS ONE.

[73] Almeida P, Katschnig D, De Boer AH (2013). HKT transporters—state of the art. Int J Mol Sci.

[74] Platten JD, Cotsaftis O, Berthomieu P, Bohnert H, Davenport RJ, Fairbairn DJ (2006). Nomenclature for HKT transporters, key determinants of plant salinity tolerance. Trends Plant Sci.

[75] Plett DC, Møller IS (2010). Na^+^ transport in glycophytic plants: what we know and would like to know. Plant Cell Environ.

[76] Rus A, Baxter I, Muthukumar B, Gustin J, Lahner B, Yakubova E (2006). Natural variants of AtHKT1 enhance Na^+^ accumulation in two wild Populations of *Arabidopsis*. PLoS Genet.

[77] Liu WH, Fairbairn DJ, Reid RJ, Schachtman DP (2001). Characterization of two HKT1 homologues from *Eucalyptus camaldulensis* that display intrinsic osmosensing capability. Plant Physiol.

[78] Ali Z, Park HC, Ali A, Oh DH, Aman R, Kropornicka A (2012). TsHKT1,2, a HKT1 homolog from the extremophile *Arabidopsis* relative *Thellungiella salsuginea*, shows K^+^ specificity in the presence of NaCl. Plant Physiol.

[79] Ali A, Park HC, Aman R, Ali Z, Yun DJ (2013). Role of HKT1 in *Thellungiella salsuginea*, a model extremophile plant. Plant Sign Behav.

[80] James RA, Davenport RJ, Munns R (2006). Physiological characterization of two genes for Na^+^ exclusion in durum wheat, *Nax1* and *Nax2*. Plant Physiol.

[81] Huang SB, Spielmeyer W, Lagudah ES, James RA, Platten JD, Dennis ES (2006). A sodium transporter (HKT7) is a candidate for *Nax1*, a gene for salt tolerance in durum wheat. Plant Physiol.

[82] Huang SB, Spielmeyer W, Lagudah ES, Munns R (2008). Comparative mapping of HKT genes in wheat, barley, and rice, key determinants of Na^+^ transport, and salt tolerance. J Exp Bot.

[83] James RA, Blake C, Byrt CS, Munns R (2011). Major genes for Na^+^ exclusion, *Nax1* and *Nax2* (wheat *HKT1,4* and *HKT1,5*), decrease Na^+^ accumulation in bread wheat leaves under saline and waterlogged conditions. J Exp Bot.

[84] Horie T, Karahara I, Katsuhara M (2012). Salinity tolerance mechanisms in glycophytes: An overview with the central focus on rice plants. Rice.

[85] Babgohari MZ, Niazi A, Moghadam AA, Deihimi T, Ebrahimie E (2013). Genome-wide analysis of key salinity-tolerance transporter (HKT1, 5) in wheat and wild wheat relatives (A and D genomes). In Vitro Cell Dev Biol-Pl.

[86] Negrão S, Almadanim C, Pires IS, Abreu IA, Maroco J, Courtois B (2013). New allelic variants found in key rice salt-tolerance genes: an association study. Plant Biot J.

[87] Amar SB, Brini F, Sentenac H, Masmoudi K, Véry AA (2014). Functional characterization in *Xenopus oocytes* of Na^+^ transport systems from durum wheat reveals diversity among two HKT1,4 transporters. J Exp Bot.

[88] Jabnoune M, Espeout S, Mieulet D, Fizames C, Verdeil JL, Conejero G (2009). Diversity in expression patterns and functional properties in the rice HKT transporter family. Plant Physiol.

[89] Yao X, Horie T, Xue SW, Leung HY, Katsuhara M, Brodsky DE (2010). Differential sodium and potassium transport selectivities of the rice OsHKT2,1 and OsHKT2,2 transporters in plant cells. Plant Physiol.

[90] Zhang JL, Flowers TJ, Wang SM (2010). Mechanisms of sodium uptake by roots of higher plants. Plant Soil.

[91] Oomen RJ, Benito B, Sentenac H, Rodríguez-Navarro A, Talón M, Véry AA (2012). HKT2, 2/1, a K^+^-permeable transporter identified in a salt-tolerant rice cultivar through surveys of natural genetic polymorphism. Plant J.

[92] Laurie S, Feeney KA, Maathuis FJM, Heard PJ, Brown SJ, Leigh RA (2002). A role for HKT1 in sodium uptake by wheat roots. Plant J.

[93] Kader MA, Seidel T, Golldack D, Lindberg S (2006) Expressions of OsHKT1, OsHKT2, and OsVHA are differentially regulated under NaCl stress in saltsensitive and salt-tolerant rice (*Oryza sativa L.*) cultivars. J Exp Bot 57:4257–4268. 10.1093/jxb/erl199 17088362

[94] Rubio F, Gassmann W, Schroeder JI (1995). Sodium-driven potassum uptake by the plant potassium transporter HKT1 and mutations conferring salt tolerance. Science.

[95] Gassmann W, Rubio F, Schroeder JI (1996). Alkali cation selectivity of the wheat root high-affinity potassium transporter HKT1. Plant J.

[96] Takahashi R, Nishio T, Ichizen N, Takano T (2007). Cloning and functional analysis of the K^+^ transporter PhaHAK2 from salt-sensitive and salt-tolerant reed plants. Biot Letter.

[97] Qiu L, Wu D, Ali S, Cai S, Dai F, Jin X (2011). Evaluation of salinity tolerance and analysis of allelic function of HvHKT1 and HvHKT2 in Tibetan wild barley. Theor App Genet.

[98] Lan WZ, Wang W, Wang SM, Li LG, Buchanan BB, Lin HX (2010). A rice high-affinity potassium transporter (HKT) conceals a calcium-permeable cation channel. Proc Natl Acad Sci U S A.

[99] Sassi A, Mieulet D, Khan I, Moreau B, Gaillard I, Sentenac H (2012). The rice monovalent cation transporter OsHKT2,4: revisited ionic selectivity. Plant Physiol.

[100] Horie T, Brodsky DE, Costa A, Kaneko T, Schiavo FL, Katsuhara M (2011). K^+^ transport by the OsHKT2,4 transporter from rice with a typical Na^+^ transport properties and competition in permeation of K^+^ over Mg^2+^ and Ca^2+^ ions. Plant Physiol.

[101] Véry AA, Sentenac H (2003). Molecular mechanisms and regulation of K^+^ transport in higher plants. Annu Rev Plant Biol.

[102] Garciadeblás B, Barrero-Gil J, Benito B, Rodríguez-Navarro A (2007). Potassium transport systems in the moss *Physcomitrella patens* : *pphak1* plants reveal the complexity of potassium uptake. Plant J.

[103] Zhang HM, Kim MS, Sun Y, Dowd SE, Shi HZ, Paré PW (2008). Soil bacteria confer plant salt tolerance by tissue-specific regulation of the sodium transporter HKT1. Mol Plant Microbe In.

[104] Wang Y, Wu WH (2013). Potassium transport and signaling in higher plants. Annu Rev Plant Biol.

[105] Waters S, Gilliham M, Hrmova M (2013). Plant high-affinity potassium (HKT) transporters involved in salinity tolerance: structural insights to probe differences in ion selectivity. Int J Mol Sci.

[106] Cao Y, Pan Y, Huang H, Jin X, Levin EJ, Kloss B (2013). Gating of the TrkH ion channel by its associated RCK protein TrkA. Nature.

[107] Vieira-Pires RS, Szollosi A, Morais-Cabral JH (2013). The structure of the KtrAB potassium transporter. Nature.

[108] Takeuchi A, Reyes N, Artigas P, Gadsby DC (2008). The ion pathway through the opened Na^+^, K^+^-ATPase pump. Nature.

[109] Doyle DA, Cabral JM, Pfuetzner RA, Kuo A, Gulbis JM, Cohen SL (1998). The structure of the potassium channel: molecular basis of K^+^ conduction and selectivity. Science.

[110] Zeng GF, Pypaert M, Slayman CL (2004). Epitope tagging of the yeast K^+^ carrier Trk2p demonstrates folding that is consistent with a channel-like structure. J Biol Chem.

