# Erratum to: Model of Cation Transportation Mediated by High-Affinity Potassium Transporters (HKTs) in Higher Plants

**DOI:** 10.1186/s12575-015-0021-y

**Published:** 2015-04-30

**Authors:** Yi Su, Weigui Luo, Wanhuang Lin, Liying Ma, Mohammed Humayun Kabir

**Affiliations:** Hunan Provincial Key Laboratory of Phytohormones and Growth Development, Hunan Agricultural University, Changsha, China; Hunan Co-Innovation Center for Utilization of Botanical Functional Ingredients, Changsha, China; Chengnan College, Hunan First Normal University, Changsha, China

## Erratum

After publication of this work [1], it has come to our attention that Mohammed Humayun Kabir’s name was displayed incorrectly. The full list of authors has now been updated. We are publishing this erratum to update the author list, which is as follows:

Yi Su, Weigui Luo, Wanhuang Lin, Liying Ma and Mohammed Humayun Kabir.

## References

[1] Su Y, Luo W, Lin W, Ma L, Kabir MH (2015). Model of Cation Transportation Mediated by High-Affinity Potassium Transporters (HKTs) in Higher Plants. Biol Proced Online.

